# Sublingual human chorionic gonadotropin as an adjuvant to ovulation induction

**DOI:** 10.5935/1518-0557.20230068

**Published:** 2024

**Authors:** Paula Almeida Galvão Ferreira, Luiz Augusto Giordano, Luiz Felipe Bittencourt

**Affiliations:** 1 Departamento de Ginecologia, Universidade Federal do Estado do Rio de Janeiro, Rio de Janeiro, RJ, Brazil; 2 Departamento de Materno-infantil, Universidade Federal Fluminense (UFF), Niterói, RJ, Brazil

**Keywords:** letrozole, clomiphene, chorionic gonadotropin, ovulation induction

## Abstract

**Objective:**

To evaluate the efficacy of sublingually administered human chorionic gonadotropin (HCG) in combination with clomiphene citrate (CC) or letrozole (LTZ) for ovulation induction.

**Methods:**

In this prospective, double-blind, randomized study, the patients were divided into two placebo groups and two intervention groups using CC, LTZ, and HCG.

**Results:**

There were no statistically significant differences in ovulation induction between the groups. We compared endometrial thickness at the beginning of the cycle and during the pre-ovulatory period, and detected a moderately positive correlation when CC was administered with HCG.

**Conclusions:**

Sublingual HCG with CC caused a moderately positive correlation with endometrial thickening when compared with that at the beginning of the cycle and during the pre-ovulatory period. There was no significant change in the number of pre-ovulatory follicles.

## INTRODUCTION

Assisted human reproduction (AR) was first performed in England in 1978, resulting in the birth of the first test-tube baby. According to data from the American Fertility Society, until the 1960s, the global infertility rate varied between 10 and 15% among the population. Currently, these levels vary between 25 and 30%. During this period, the assessment and treatment of infertility have undergone a dramatic transformation (de Moura *et al*., 2009).

Three major factors have greatly impacted the observed changes in infertility assessment and therapy. The first was the introduction of *in vitro* fertilization (IVF) and other AR techniques. These techniques have enabled the in-depth examination of the reproductive processes, thereby improving the prognosis of numerous infertile couples. Furthermore, owing to social changes, more women are now attempting to conceive at an advanced age when fertility is inherently reduced. Other factors include advances in AR and concerns regarding age-related decline in fertility. Overall, these factors have afforded greater media attention and public awareness (Fritz & Speroff, 2015).

The availability of AR services has dramatically increased over the past 25 years. Physicians are more aware of infertility and are better trained to assess and treat underlying causes. Infertility has become more apparent and socially acceptable, couples are less reluctant to seek evaluation and treatment (Dzik *et al*., 2012).

However, the demand for treatment, even in developed countries, remains far from ideal. For example, only 65% of infertile couples in the United States seek medical care (Richmond *et al*., 2005). The choice of treatment must be tailored to the specific infertile couple. To individualize the treatment options, the underlying cause of infertility, the severity of the causal factor, the female’s age; and ovarian reserve must be evaluated, along with factors that may influence clinical decisions regarding the treatment offered to the couple. In addition, religion, success rate, financial aspects, and a couple’s anxiety can impact the decision regarding the type of treatment employed.

The main causes of marital infertility are ovulatory factors, and the underlying causes of anovulation tend to vary. Thyroid disorders, hyperprolactinemia, adrenal disease, pituitary or ovarian tumors, eating disorders, polycystic ovary syndrome (PCOS), and obesity are all commonly associated with ovulation dysfunction (Fritz & Speroff, 2015).

To treat females experiencing anovulation, ovulation induction with scheduled intercourse or intrauterine insemination using clomiphene citrate (CC) and aromatase inhibitors, such as letrozole (LTZ), are common practices at infertility clinics. Compared with other AR procedures, ovulation induction is less invasive, of lower-cost, and a less complex treatment strategy. In cases of insufficient response, other drug options, such as exogenous gonadotropins, can be considered. Moreover, success rates increase annually (Filicori *et al*., 2002; Fritz & Speroff, 2015).

Ovulation disorders are responsible for female infertility in 40% of cases; and 15% of all infertility cases; therefore, it remains an extremely relevant factor. Accordingly, low-complexity reproductive treatments are commonly employed. In the case of ovulation disorder-related infertility, the therapeutic option of choice is ovulation induction using CC. PCOS is the most common cause of chronic anovulation; however, a conclusive diagnosis of these alterations can be challenging. According to the Rotterdam Consensus, the primary investigation method, the patient must present with at least two of the following criteria: oligo/amenorrhea, clinical and/or laboratory hyperandrogenism, and polycystic ovarian morphology on ultrasound. PCOS exhibits a broad clinical presentation and it is typically associated with insulin resistance in addition to its typical manifestations (Fritz & Speroff, 2015).

CC was synthesized in 1956 and was introduced into clinical trials in 1960. Since then, it has remained the first choice for treating anovulation in reproductive medicine (Jirge & Patil, 2010).

Aromatase inhibitors are primarily used for treating breast cancer. However, these agents were recently found to be effective ovulation inducers (Jirge & Patil, 2010).

In 2018, a study showed that LTZ could be the first-line pharmacological treatment for ovulation induction in patients with PCOS, owing to the lower risk of multiple pregnancies with LTZ than with CC.

In reports published in 2002 and 2004, human chorionic gonadotropin (HCG), in association with follicle-stimulating hormone (FSH), was shown to potentiate the final development of ovarian follicles (Filicori *et al*., 2002).

Given the evidence supporting the effectiveness of HCG in ovarian stimulation and observed benefits in reproductive therapy, further research is warranted to conclusively establish the application of this agent. In addition, novel concepts may arise, such as the development of alternate routes of administration, considering that administering injections can be inconvenient, painful, and cause loss of patient compliance to treatment. The use of sublingual HCG for ovulation induction may represent a new perspective on reproductive treatment.

The objective of the study was to analyze whether the association of sublingual HCG with CC and letrozole modifies the results of the number of follicles, endometrial thickening and pregnancy rate in the studied groups.

## MATERIALS AND METHODS

Type of Study and Place of Completion

This prospective, triple-blind, randomized study was conducted at the University Hospital Gaffrée and Guinle (HUGG) from August 2019 to December 2020.

The study protocol was approved by the Research Ethics Committee of the institution. All the patients agreed to participate and signed an informed consent form.

### Patients

Initially, 125 patients were selected for study participation and treated at the HUGG Infertility Clinic. Of these, 63 met the inclusion criteria.

### Inclusion Criteria

Patients diagnosed with infertility and with indications for treatment with ovulation induction and programmed intercourse.

Age ≤38 years;

Normal ovarian reserve (evaluated with the antral follicle count (greater than 7) and FSH dosage (greater than 10 mUI/ml);

With at least one patent tube;

Partner with satisfactory seminal analysis (According to WHO criteria).

### Exclusion Criteria

Decompensated systemic diseases;

Whose partner presented seminal alterations necessitating high complex AR

Patients previously submitted to FIV.

### Drugs Used

CC 50 mg: The options used were Clomid (Medley Laboratory) and Indux (EMS Laboratory). The medication was purchased by the patient.

LTZ 2.5 mg: Manufactured and distributed in Brazil by the Eurofarma Pharmaceutics. The medication was purchased by the patient.

HCG 200 IU: Produced by Pharmacy Manipulation Pharmactive, under authorization from the National Health Surveillance Agency to operate, as provided in Resolution RE-N⁰ 3,727, on August 19, 2011; this was funded by the researcher.

Placebo: The placebo was produced by Farmácia de Manipulação Pharmactive, with authorization from the National Health Surveillance Agency to operate, as provided in Resolution RE-N⁰ 3,727 on August 19, 2011.

All adverse drug effects were followed up and treated.

### Interventions

Enrolled patients underwent consultation, including an assessment of medical history and physical examination, and tests involved in the basic infertility workup were analyzed. In addition, a spermogram, hysterosalpingography, transvaginal ultrasound, and hormone levels (prolactin, thyroid stimulating hormone [TSH], FSH, luteinizing hormone [LH], and progesterone) were evaluated. We also completed a socioeconomic questionnaire.

**Figure 1:**
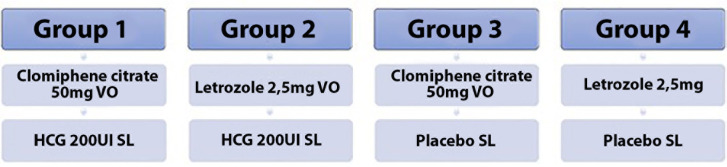
Research groups and medications used.

The patients were divided using the random sequence generator portal (https://www.random.org/sequences/) into two intervention groups and two placebo groups as follows (Figure 1):

Group 1 - CC and HCG;

Group 2 - LTZ and HCG;

Group 3 - CC and placebo;

Group 4 - LTZ and placebo.

After randomization, each patient received an envelope with a research registration number. Each envelope contained the CC or LTZ prescription and HCG or placebo pills. The placebo and HCG pills were identical and could not be distinguished based on appearance. These compounds were prepared at compounding pharmacies.

Investigating physicians and study participants were unaware of the designated grouping at the time of the statistical analysis.

Group 1: Patients orally administered CC (50 mg) from days 3 to 7 of the cycle, initiating sublingual HCG (200 IU/day) when the mean diameter of the largest follicle reached 14 mm on ultrasound. In 2004, a new study was carried out on the subject, with the prescription of HCG in the final phase of follicular development. At the time, 200 IU per day was prescribed.

Group 2: Patients orally administered LTZ (2.5 mg) from days 3 to 7 of the cycle, subsequently initiating sublingual HCG (200 IU/day) when the mean diameter of the largest follicle reached 14 mm on ultrasound.

Group 3: Patients orally administered CC (50 mg) from days 3 to 7 of the cycle, initiating the sublingual placebo when the mean diameter of the largest follicle reached 14 mm on ultrasound.

Group 4: Patients orally administered LTZ (2.5 mg) from days 3 to 7 of the cycle, initiating the sublingual placebo when the mean diameter of the largest follicle reached 14 mm.

Herein, we examined the following parameters: endometrial thickness at the beginning of the cycle, endometrial thickness during the pre-ovulatory period, number of pre-ovulatory follicles, and pregnancy rate.

**Figure 2:**
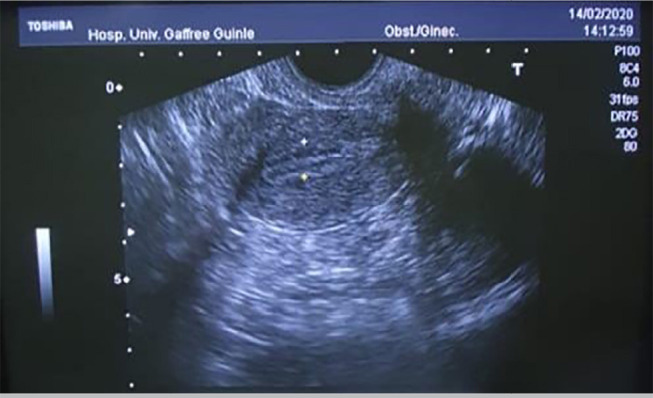
Measurement of the trilaminar endometrium.

To analyze the endometrium and ovaries, all the patients underwent baseline transvaginal ultrasound on day 2 of the menstrual cycle. This served as a reference for comparing and observing possible changes caused by the medications used in the study. An ultrasound was then performed on day 7 or 8 of the cycle; subsequently, additional examinations were repeated at two-day intervals until ovulation was confirmed. For each ultrasound evaluation, the number and mean diameter of follicles, as well as the thickness and morphology of the endometrium, were evaluated. When counting the number of pre-ovulatory follicles, only follicles with a mean diameter of ˃16 mm were included. To assess the endometrium, the distance between the two endometrial interfaces at the point of the greatest thickness was considered (Figure 2). The ovulatory cycle was considered: visualization of follicular growth and subsequent disappearance with the characteristic formation of the corpus luteum, presence of free fluid posterior to the uterus, and changes in the endometrial pattern from proliferative to secretory phases. All examinations were performed by a team of three examiners, previously trained and supervised. The examinations were performed using a single device (GE brand, Voluson S6 model).

Beta-HCG was administered between 24 and 48 h after the last sublingual HCG tablet to assess the serum HCG level.

A new beta-HCG dose was administered 14 days after ovulation detection to determine the pregnancy rate.

Patients who could not become pregnant remained under care for an additional period to establish alternate infertility treatments.

### Ethics and authorization

The study was carried out with the approval of the Research Ethics Committee of this institution. All the patients agreed and signed the informed consent form. HCG Placebo Produced by Farmácia de Manipulação Pharmactive, with authorization from the National Health Surveillance Agency for operation, as provided in Resolution RE-N⁰ 3727, of August 19, 2011.

### Statistical analysis

The sample size calculation revealed that the ideal number of patients to obtain statistically relevant results is 61 patients. However, during the period in which this study was carried out, only 51 patients successfully completed the induction cycle. In addition, of the 125 patients who initially responded to the questionnaire, 62 had some exclusion criteria and 12 did not respond to the prescribed treatment.

The normalization of the diameter distribution was analyzed using the nonparametric Kolmogorov-Smirnov test. Normally distributed data are expressed as mean and standard deviation; otherwise, they are presented as median and interquartile range.

To analyze the groups in relation to follicular growth and endometrial thickening, we performed parametric tests, including univariate analysis of variance (ANOVA) with Tukey’s post-test. However, when the data were not normally distributed, the Kruskal-Wallis test with Dunn’s post-test was performed.

The Chi-square test, a nonparametric test that assesses the existence of an association between qualitative variables, was used to analyze the pregnancy rate (yes or no) in groups.

To analyze the endometrium before and after drug-induced stimulation within the same group, the paired t-test or Wilcoxon test was used. However, to assess differences between groups, we used the variation of these parameters (thickness after - thickness before) and subsequently performed univariate ANOVA or Kruskal-Wallis test.

To analyze the endometrium before and after drug-induced stimulation, we performed Pearson’s correlation test, which assesses the level of association between two continuous variables, regardless of the normality of the sample.

To reduce pharmacological standardization bias, a quantitative beta-HCG test was performed in all patients 24-48 h after the last sublingual HCG tablet. Therefore, the same statistical tests were performed, but patients in the LTZ with HCG and CC with HCG groups who presented a negative beta-HCG result were excluded.

The significance level was set at *p*<0.05. All data were analyzed using the SPSS version 20.0 software (IBM Corp., Armonk, NY, USA).

## RESULTS

All invited and eligible female subjects agreed to participate in the present study. Sixty-two patients were excluded for the following reasons: 10 due to bilateral tubal obstruction, 12 were ˃ 38 years old, 5 had previously undergone highly complex treatments, 4 presented with deep endometriosis, 6 had partners presenting seminal alterations that would prevent spontaneous pregnancy, and 3 owing to decompensated clinical diseases; all these cases met the established exclusion criteria. All excluded patients continued follow-ups at the infertility clinic. In addition, 22 patients were excluded owing to treatment abandonment.

Of the 63 enrolled patients, ovulation induction cycles were canceled in 12 patients as they failed to respond to ovulation inducers. Of these, 6 did not respond to LTZ, and 6 failed to respond to CC. In both cases, alternate medications were prescribed to induce ovulation.

Overall, 51 patients completed the treatment cycle proposed in the present study protocol and were assigned to four groups. The first group comprised 13 patients who received CC with sublingual HCG. The second group comprised 11 patients who received LTZ with sublingual HCG. The third group comprised 15 patients who received CC with a placebo. The fourth group comprised 12 patients who received CC with a placebo.

**Figure 3:**
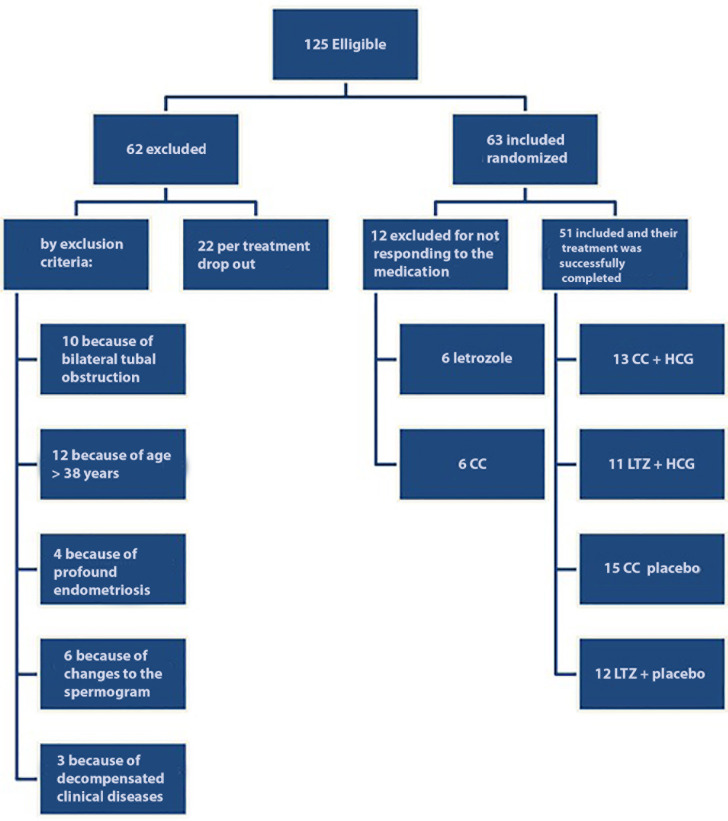
Process of exclusion and inclusion of patients.

The results described above are shown in Figure 3.

## Sample characterization

In the present study, the mean age of patients was 32.5 years. Considering family income, the average family had two minimum wages. The average years of schooling during the study period was 12 years. Of the 51 patients included in this study, 88.2% experienced primary infertility. The patients had attempted to get pregnant for an average of 3.5 years before seeking specialized treatment. The mean body mass index (BMI) was 28.4, with a standard deviation of 5.41. Table 1 summarizes partial patient information.

**Table 1:** Sample characterization.

Variable	Mean	Median	SD	Maximum	Minimum
Age	32.5	32	2.99	37	26
Years of study	12	13	2.18	17	8
Income	2.12	2	0.99	5	<1
Time	3.56	3	2.17	10	1
BMI	28.4	29.4	5.41	40	18.7
W/H	0.76	0.8	0.12	0.96	0.5

SD, standard deviation; BMI, body mass index; W/H, waist-to-hip ratio.

We analyzed the following parameters in the present study: endometrial thickness at the beginning of the menstrual cycle, endometrial thickness during the pre-ovulatory period, number of pre-ovulatory follicles, and pregnancy rate.

Table 2 shows the effects of treatment on the number of pre-ovulatory follicles and endometrial thickening. No significant differences were observed between examined groups.

**Table 2:** Effect of treatments on the number of pre-ovulatory follicles and endometrial thickening.

	Clomiphene (n=15)	Clomiphene + HCG (n=13)	Letrozole (n=12)	Letrozole + HCG (n=11)	*p*-value
Number of pre-ovulatory follicles	2.07±1.22	2.23±1.01	1.50±0.52	1.72±0.90	0.243
Endometrial thickening	9.25±2.98	9.93±3.57	8.08±1.71	9.68±2.55	0.394

Data are presented as the mean and standard deviation; one-way ANOVA test.

HCG, human chorionic gonadotropin.

There was no significant association between groups in terms of pregnancy rates. Thus, the pregnancy rates were similar between the established groups (Table 3).

**Table 3:** Effect of treatments on pregnancy rate.

	Clomiphene (n=15)	Clomiphene + HCG (n=13)	Letrozole (n=12)	Letrozole + HCG (n=11)	*p*-value
Yes	3	2	3	2	0.944
No	12	11	9	9	

Chi-square test.

HCG, human chorionic gonadotropin.

Table 4 presents the endometrial analysis in relation to its thickness at the beginning of the menstrual cycle and during the pre-ovulatory period after drug-induced stimulation. Considering all groups, endometrial thickness was increased during the pre-ovulatory period. Endometrial growth between the groups was evaluated based on analyzing variations in thickness. Despite the LTZ group exhibiting an endometrial thickness 35.5% less than the CC group, the difference was not statistically significant.

**Table 4:** Effects of treatments on the endometrium: comparison of endometrial thickening at the beginning of the cycle with that during the pre-ovulatory period.

	Begin	Pre-ovulatory	p-value (before e after)	Variation
Clomiphene (n=15)	4.33±1.47	9.25±2.99	<0.0001^*^	4.92±2.78
Clomiphene + HCG (n=13)	5.07±1.75	9.93±3.58	<0.0001^*^	4.86±2.94
Letrozole (n=12)	4.92±1.28	8.08±1.72	<0.0001^*^	3.17±2.36
Letrozole + HCG (n=11)	5.13±1.54	9.68±2.55	<0.0001^*^	4.55±2.51
*p*-value variation	-	-	-	0.365

Data are expressed as mean and standard deviation.

* represents a significant difference (paired t-test); for variation, a one-way ANOVA test was used.

HCG, human chorionic gonadotropin.

Table 5 presents the association between endometrial thickness before and after drug-induced stimulation. At the 95% significance level, we detected a moderately positive correlation between endometrial thickness and treatment with CC combined with HCG. In addition, the group taking CC with the placebo showed a positive but weak correlation. There were no statistically significant differences noted in other groups.

**Table 5:** Effects of treatments on the endometrium: comparison of endometrial thickening at the beginning of the cycle with that during the pre-ovulatory period.

Group	Pearson’s correlation	*p*-value
CC+HCG	0.574	0.040^*^
LTZ+HCG	0.331	0.319
CC+PLAC	0.382	0.047^*^
LTZ+PLAC	-0.227	0.479

Statistical significance of 95%; SD: standard deviation.

CC, clomiphene citrate; HCG, human chorionic gonadotropin; LTZ, letrozole; PLAC, placebo.

After treatment completion, quantitative beta-HCG was administered 24 to 48 h after the last sublingual HCG tablet to assess the serum level of the medication. The results are presented in Table 6.

**Table 6:** Number of patients with positive serum beta-HCG levels after sublingual HCG administration.

CC + HCG	LTZ + HCG	CC + placebo	LTZ + placebo
6	5	0	0

CC, clomiphene citrate; HCG, human chorionic gonadotropin; LTZ, letrozole; PLAC, placebo.

Statistical tests were applied similarly, excluding patients with beta-HCG negativity in the HCG groups.

Univariate ANOVA was used to compare the number of follicles and endometrial thickening for independent groups with normal distribution. Table 7 demonstrates the treatment effects on the number of pre-ovulatory follicles and endometrial thickening. No significant differences were observed between the examined groups.

**Table 7:** Effect of treatments on the number of pre-ovulatory follicles and endometrial thickening.

	Clomiphene (n=15)	Clomiphene + HCG (n=7)	Letrozole (n=12)	Letrozole + HCG (n=5)	*p*-value
Number of pre-ovulatory follicles	2.07±1.22	2.57±0.787	1.50±0.52	1.92±1.02	0.133
Endometrial thickening	9.24±2.98	9.23±4.83	8.08±1.71	8.91±3.00	0.743

Data are presented as the mean and standard deviation; one-way ANOVA test.

HCG, human chorionic gonadotropin

There was no significant association between the groups in terms of pregnancy rates. Thus, the pregnancy rates were similar between the examined groups (Table 8).

**Table 8:** Effect of treatments on pregnancy rate.

	Clomiphene (n=15)	Clomiphene + HCG (n=7)	Letrozole (n=12)	Letrozole + HCG (n=5)	*p*-value
Yes	3	1	3	1	0.946
No	12	6	9	5	

Chi-square test

HCG, human chorionic gonadotropin.

Table 9 presents the endometrial thickness at the beginning of the menstrual cycle and during the pre-ovulatory period after drug-induced stimulation. For all groups, endometrial thickness was greater during the pre-ovulatory period than that at the beginning of the menstrual cycle. However, on analyzing endometrial growth between the groups, there was no significant difference based on evaluating these variations (Table 10).

**Table 9:** Effects of treatments on the endometrium: comparison of the beginning of the cycle with the pre-ovulatory period.

	Begin	Pre-ovulatory	*p*-value (before e after)	Variation
Clomiphene (n=15)	4.33±1.47	9.25±2.99	<0.0001	4.92±2.78
Clomiphene + HCG (n=13)	4.60±1.59	9.93±4.83	<0.0001	4.63±4.01
Letrozole (n=12)	4.92±1.28	8.08±1.72	<0.0001	3.17±2.36
Letrozole + HCG (n=11)	4.62±1.46	9.35±3.00	<0.0001	4.53±2.62
p-value variation	-	-	-	0.653

Data are expressed as mean and standard deviation.

* represents a significant difference (paired t-test); for variation, a one-way ANOVA test was used.

**Table 10:** Effects of treatments on the endometrium: comparison of the beginning of the cycle with the pre-ovulatory period.

Group	Pearson’s Correlation	*p*-value
CC+HCG	0.636	0.038^*^
LTZ+HCG	0.464	0.354
CC+PLAC	0.382	0.160
LTZ+PLAC	-0.227	0.479

Statistical significance of 95%; SD: standard deviation.

As shown in Table 10, at a significance level of 95%, we detected a strong positive correlation between endometrial thickness and the treatment with CC combined with HCG. As shown above, there were no statistically significant differences in other groups.

## DISCUSSION

CC was first used as an ovulation inducer in 1960, and since then has gained importance in AR treatment strategies. However, the presence of insufficient endometrial thickening, as well as the decrease in cervical mucus exhibited by a subsection of CC-treated patients, has motivated the search for novel treatment alternatives.

LTZ, an aromatase inhibitor, has emerged as a promising therapeutic agent, given that in addition to ovulatory stimulation, LTZ exhibits lower antiestrogenic effects on the endometrium and cervical mucus than CC^24^. However, potential teratogenicity has instigated the search for novel treatment agents.

Subcutaneous HCG (200 IU/day) is reportedly administered during the final follicular phase. HCG was found to demonstrate a similar potential for FSH to stimulate ovulation but at a lower cost (Filicori *et al*., 2004).

To the best of our knowledge, there are no available reports on the sublingual use of 200 IU HCG for ovulation induction. Accordingly, we hypothesized that sublingual HCG could induce a similar effect to that of the subcutaneous HCG. The use of HCG in association with CC and LTZ, consolidated for treating anovulation, could potentiate the beneficial effects on ovulation induction.

In the present study, sublingual HCG tablets were developed at a compounding pharmacy, with authorization from the National Health Surveillance Agency, as provided in Resolution RE-N⁰ 3,727, on August 19, 2011 (Brazil, 2011). The drug was also produced under the supervision of the same pharmacist responsible for the company’s technique. This strategy aimed to reduce pharmacological standardization bias, given that sublingual HCG is currently not manufactured by large pharmaceutical companies.

Compared with injectable routes, sublingual HCG has advantages such as easy administration and absence of pain upon application, in addition to the low cost of medication, thereby increasing treatment compliance among patients.

In 2019, a Brazilian study examined the stability of solution samples containing HCG as an active ingredient administered intramuscularly, orally, and sublingually. The authors observed that the concentration of the active ingredient was not altered at different temperatures, maintaining its efficacy (Barbosa *et al*., 2019). Accordingly, the concentration of the active ingredient was not influenced by tablet transport and consequent temperature fluctuations.

Given that we could not assess the active ingredient concentration in each tablet, we measured the serum beta-HCG 24-48 h after ingestion of the last sublingual HCG. The same laboratory and test kits were employed to assess beta-HCG in all patients. An additional statistical evaluation was performed considering only patients with positive beta-HCG, with no statistical difference noted in the number of pre-ovulatory follicles, endometrial thickening, and pregnancy rates; however, the sample size may have influenced this result.

Herein, participating patients were recruited from the HUGG Infertility Clinic. As this is a subspecialty outpatient clinic in a tertiary hospital with referred patients that met specific criteria for follow-up, the uptake during the study period was limited. In addition, the coronavirus disease pandemic impacted patient selection for research purposes. The sample size calculation revealed that the ideal number of patients to obtain statistically relevant results was 61. However, only 51 patients successfully completed the induction cycle during the study period. In addition, of the 125 patients who initially responded to the questionnaire, 62 met the exclusion criteria, and 12 did not respond to the prescribed treatment.

The stratification of patients into four groups was based on the comparison of sublingual HCG, an innovative treatment, with two consolidated treatments that induce ovulation, i.e., CC and LTZ. However, this stratification resulted in a reduced sample size from each group, consequently impacting the statistical significance of the study. Further studies with larger sample sizes should be conducted to support these findings.

CC and LTZ tablets were made available to the patients and administered without supervision, which could induce a confounding factor. However, prescriptions and a list of guidelines were used for controlling this factor, in addition to in-person follow-up every two days at the HUGG Infertility Clinic during medication use.

To assess follicular growth and endometrial thickening, we performed transvaginal ultrasound imaging, the test of choice for assessing ovulation induction cycles. Moreover, transvaginal ultrasound is a low-cost technique and does not expose the patient to ionizing radiation. Despite being the most suitable method for this purpose, transvaginal ultrasound has disadvantages, such as patient discomfort during the procedure and the need for a skilled examiner. In addition, given the dynamic nature of this examination, there may be intraand inter-observer variations.

Considering the number of pre-ovulatory follicles, our findings revealed no statistically significant difference between groups when comparing LTZ and placebo with LTZ and HCG, CC and placebo with LTZ and placebo, CC and placebo with CC and HCG, and CC and HCG with LTZ and HCG. However, it has been reported that CC can induce a greater number of follicles than LTZ (Pavone & Bulun, 2013). Therefore, we speculate whether the failure to obtain a notable difference in follicles between the CC with placebo and LTZ with placebo groups could be attributed to the sample size and whether statistical significance could be detected in other groups by correcting for this factor.

Compared with CC, one advantage of LTZ is the superior stimulation of endometrial thickening (Pavone & Bulun, 2013). However, the statistical significance of this variable was not verified in the present study.

Furthermore, we compared endometrial thickness at the beginning of the cycle and during the pre-ovulatory period for each group and detected a moderately positive correlation when CC was taken with HCG. Comparing variables in the CC combined with placebo group, we detected a weak positive correlation with endometrial thickening. Given the known deleterious effect of CC on the endometrium (Pavone & Bulun, 2013), the association with HCG may afford a new approach for patients with insufficient endometrial thickening.

In a Cochrane review, LTZ-treated females were shown to exhibit higher rates of clinical pregnancy and live births than CC-treated subjects (Franik *et al*., 2018).

However, this result was not reproduced in the present study. Performing only one ovulation induction cycle alone may have contributed to this discrepancy, given that a pregnancy rate of 15% has been documented in young patients after a single induction cycle (Franik *et al*., 2018).

As noted, there was no statistical significance in relation to the number of follicles and pregnancy rate in all groups studied; however, new studies with sublingual HCG, with a larger sample size, should be performed to elucidate these issues.

Considering only beta-HCG-positive patients after sublingual HCG, a refined analysis of the groups presented similar results to those without refinement.

## CONCLUSIONS

Considering the investigated population, we can conclude that:

Sublingual HCG combined with CC was positively correlated to endometrial thickening when compared with the beginning of the cycle and during the pre-ovulatory period. Therefore, combined treatment with HCG may represent a promising strategy for ovulation induction, especially in cases of insufficient endometrial thickening. There was no statistically significant difference in the LTZ with placebo and LTZ with HCG groups.

Furthermore, we detected no statistically significant difference in the number of follicles or pregnancy rates among the examined groups.

Additional studies are warranted to assess sublingual HCG in a larger patient population to corroborate the findings of the present study.
